# Genetic mapping of stripe rust resistance in a geographically diverse barley collection and selected biparental populations

**DOI:** 10.3389/fpls.2024.1352402

**Published:** 2024-07-19

**Authors:** Davinder Singh, Laura Ziems, Mumta Chettri, Peter Dracatos, Kerrie Forrest, Sridhar Bhavani, Ravi Singh, Charles W. Barnes, Patricio Javier Noroña Zapata, Om Gangwar, Subodh Kumar, Subhash Bhardwaj, Robert F. Park

**Affiliations:** ^1^ Plant Breeding Institute, School of Life and Environmental Sciences, University of Sydney, Cobbitty, NSW, Australia; ^2^ La Trobe Institute of Sustainable Agriculture & Food (LISAF), Department of Animal, Plant and Soil Sciences, AgriBio, Bundoora, VIC, Australia; ^3^ Agriculture Victoria, AgriBio, Centre for AgriBioscience, Cobbitty, VIC, Australia; ^4^ Global Wheat Program, International Maize and Wheat Improvement Center, Texcoco, Mexico; ^5^ Instituto Nacional de Investigaciones Agropecuarias (INIAP), Quito, Ecuador; ^6^ Forest Health Protection – Region 5, USDA Forest Service, San Bernardino, CA, United States; ^7^ ICAR-Indian Institution of Wheat and Barley Research, Regional Station, Flowerdale, Shimla, India

**Keywords:** barley, yellow rust, *Puccinia striiformis* f. sp. *hordei*, GWAS, mapping, QTL, marker assisted selection

## Abstract

Barley stripe or yellow rust (BYR) caused by *Puccinia striiformis* f. sp. *hordei* (*Psh*) is a significant constraint to barley production. The disease is best controlled by genetic resistance, which is considered the most economical and sustainable component of integrated disease management. In this study, we assessed the diversity of resistance to *Psh* in a panel of international barley genotypes (n = 266) under multiple disease environments (Ecuador, India, and Mexico) using genome-wide association studies (GWASs). Four quantitative trait loci (QTLs) (three on chromosome 1H and one on 7H) associated with resistance to *Psh* were identified. The QTLs were validated by mapping resistance to *Psh* in five biparental populations, which detected key genomic regions on chromosomes 1H (populations Pompadour/Zhoungdamei, Pompadour/Zug161, and CI9214/Baudin), 3H (Ricardo/Gus), and 7H (Fumai8/Baronesse). The QTL *RpshQ.GWA.1H.1* detected by GWAS and *RpshQ.Bau.1H* detected using biparental mapping populations co-located were the most consistent and stable across environments and are likely the same resistance region. *RpshQ.Bau.1H* was saturated using population CI9214/Baudin by enriching the target region, which placed the resistance locus between 7.9 and 8.1 Mbp (flanked by markers *sun_B1H_03*, 0.7 cM proximal to *Rpsh_1H* and *sun_B1H_KASP_02*, 3.2 cM distal on 1HS) in the Morex reference genome v.2. A Kompetitive Allele Specific PCR (KASP) marker *sun_B1H_KASP_01* that co-segregated for *RpshQ.Bau.1H* was developed. The marker was validated on 50 Australian barley cultivars, showing well-defined allelic discrimination and presence in six genotypes (Baudin, Fathom, Flagship, Grout, Sakurastar, and Shepherd). This marker can be used for reliable marker-assisted selection and pyramiding of resistance to *Psh* and in diversifying the genetic base of resistance to stripe rust.

## Introduction

Stripe or yellow rust caused by *Puccinia striiformis* f. sp. *hordei* (*Psh*) is a fungal disease that affects barley production significantly by reducing yield and grain quality. *Psh* has not yet been detected in Australia and poses a serious exotic pathogen threat, especially considering that Australian barley germplasm has shown a high frequency of susceptibility when tested at the International Maize and Wheat Improvement Centre (CIMMYT) Mexico (Wellings et al., 2000; Derevnina et al., 2015). Genetic resistance is the most cost-effective and sustainable component of integrated disease management. Both qualitative resistance and quantitative resistance to *Psh* have been reported (Nover and Scholz, 1969; Chen and Line, 1999; Dracatos et al., 2019), although significantly fewer resistance genes have been formally designated for barley stripe rust than other barley rusts. Conventional resistance to stripe rust in barley is governed by seedling resistance genes that are race specific and have been rendered ineffective in many geographical areas where stripe rust is extant. Very few studies have characterised partial or adult plant resistance (APR) to stripe rust in barley. APR is esteemed for its value in contributing to race non-specific and durable resistance as established in the case of wheat stripe rust. Several recessive (*rps*) and dominant (*Rps*) genes (catalogued or provisionally designated) have been identified over the past 40 years (Clare et al., 2016). To date, however, only seven genes have been genetically mapped: *rps1* on chromosome 3H (Yan and Chen, 2007); *Rps4* on 1H (Johnson, 1968); *rps5* on 4H (Esvelt Yan and Chen, 2006; Klos et al., 2016); *Rps6* on 7HL (Dawson et al., 2016); *Rps7* and *Rps8* on 1H and 4H, respectively (Bettgenhaeuser et al., 2021); and *Rps9* on 5H (Clare et al., 2016). Many of these genes have been rendered ineffective with the detection of new races of the pathogen. Quantitative trait loci (QTLs) conferring resistance to several formae speciales of *P. striiformis* were recently identified through genome-wide association studies (GWASs) of barley collections (Vatter et al., 2018; Verma et al., 2018; Visioni et al., 2018) and linkage mapping of biparental populations (Toojinda et al., 2000; Castro et al., 2003; Derevnina et al., 2015). Most of these studies were confined to the identification of QTL without further validation, characterisation, mapping, or development of linked markers, limiting the efficient utilisation of the identified resistance in breeding and marker-assisted selection.

A previous study performed by Singh et al. (2018) assembled an international barley panel of 282 lines (from 26 countries) carrying various levels of field resistance to barley leaf rust (BLR). GWAS on this panel identified 13 QTLs significantly associated with resistance to BLR at adult plant growth stages. We hypothesised that this panel, which carries rich diversity of BLR resistance, may also carry useful stripe rust alleles. This is based on our experience in wheat where partial APR genes have been found to be pleiotropic (effective against multiple pathogens), a theory well exemplified by three wheat APR genes, *Lr34/Yr18/Sr57/Pm38*, *Lr46/Yr29/Sr58*, and *Lr67/Yr46/Sr55*, conferring resistance to multiple rust pathogens (Park, 2018).

In the present study, we assessed the diversity of stripe rust resistance in a subset of the Singh et al. (2018) international barley panel (n = 266) under three disease environments and performed GWAS to identify genomic regions associated with resistance to *Psh* populations prevalent in Ecuador, India, and Mexico. In addition, these studies systematically i) validated stripe rust QTL identified via GWAS using five biparental mapping populations, ii) mapped the most stable genomic region on chromosome 1H associated with resistance to stripe rust, and iii) developed closely linked markers for the mapped 1H locus.

## Materials and methods

### Plant material

An international panel comprising 266 diverse barley entries from 26 countries (**Supplementary File S1**) was used for GWAS. Of the 266 lines, 17% originated from Africa, 4% from Asia, 43% from Europe, 2% from the Middle East, 7% from North America, 22% from Oceania, and 4% from South America, and 1% were of unknown origin. Five biparental recombinant inbred line (RIL) F_7:8_ populations [Pompadour/Zhoungdamei (n = 89), Pompadour/Zug161 (n = 80), Fumai8/Baronesse (n = 84), Ricardo/Gus (n = 78), and CI9214/Baudin (n = 73)] were used for QTL mapping. These populations were chosen because at least one of the parents in each showed a resistant response to stripe rust. Fifty Australian barley cultivars (**Supplementary File S2**) were used as a validation set and genotyped with developed Kompetitive Allele Specific PCR (KASP) markers.

### Phenotypic evaluation

The barley panel was assessed for BYR at the Instituto Nacional de Investigaciones Agropecuarias (INIAP), Ecuador (2017); CIMMYT, Toluca, Mexico (2019 and 2020); and Indian Council of Agricultural Research Centre (ICAR) Flowerdale Research Centre, Shimla, India (2018). The five RIL populations (for mapping) and a set of 50 Australian barley cultivars (for marker validation) were assessed at CIMMYT, Mexico, in a single year (either 2018 or 2020).

At INIAP, Ecuador, the experimental material was sown in blocked groups (1 × 1 m with 30-cm inter-block space). Each block comprised six equally spaced rows (1 m), each representing one test line. Five blocks were sown between and perpendicular to the susceptible spreader rows. The spreaders contained equal parts of the stripe rust susceptible varieties Shyri 89 and Shyri 2000. Spreader rows were infected by naturally occurring *Psh* inoculum. The field plots at CIMMYT, Mexico, comprised 1-m paired rows sown on top of 0.8-m-wide raised beds. The susceptible spreader variety Apizaco 36 was sown as hill plots in the middle of the 0.5-m-wide alleys on one side of each test plot. Greenhouse-increased fresh urediniospores of the Mexican variant of *Psh* race 24 [PshMEX-1 , virulent on stripe rust differentials Topper (no known gene), Cambrinus (*Rps4*), Mazurka (*Rps1.c*), Varunda (*rpsVa1* and *rpsVa2*), Emir (*rpsEm1* and *rpsEm2*), Heils Franken (*Rps4* and *rpsHF*), Abed Binder (*rps2*), and Trumpf (*rpsTr1* and *rpsTr2*), and avirulent on Bigo (*Rps1.b*) and I 5 (*rps3* and *rps15*) and the bread wheat cultivar Morocco] were suspended in Soltrol 170 oil and sprayed onto ~1-month-old spreaders. At ICAR, India, each panel line was planted as a single 1-m row. To ensure the uniformity of stripe rust infection and maintenance of high disease pressure, a local susceptible line (Barley local) was added as a disease spreader after every 20 lines. Two bordering rows of the susceptible line were sown on all the sides of the panel. Stripe rust inoculations were performed with a mixture of the most predominant and virulent pathotypes just at the emergence of the flag leaf. Fresh urediniospores drawn from a fortnight-old culture were suspended in Soltrol 170 and spray inoculated on spreader rows with the help of atomisers.

At all sites, disease severity was recorded according to a modified Cobb scale by recording disease severity (DS%) and host response (R, MR, MR, MS, and S) (Peterson et al., 1948) when the severity on the respective susceptible check varieties reached 100%. Disease coefficient of infection (CI) was derived from the product of DS and fixed coefficient (0.20, 0.40, 0.6, 0.8, and 1.0 denoting R, MR, MR-MS, MS, and S, respectively) for resistance ratings and QTL mapping. Based on CI, lines were classified into five categories [R = resistant (0–20), MR = moderately resistant (21–40), MR-MS = moderately resistant to moderately susceptible (41–60), MS = moderately susceptible (61–80), and S = susceptible (81–100)].

Descriptive statistics and histogram visualisation of CI for an international panel at each site and each RIL population were performed using base R (R Core Team, 2020). Correlation coefficients between international panel field sites were performed using the R package “Hmisc” from Harrell (2023).

### Genotyping of international panel and RILs

Genomic DNA extraction was described previously by Singh et al. (2018). DNA samples from the 266 lines and progeny and parents from each of the five biparental mapping populations (Pompadour/Zhoungdamei, CI9214/Baudin, Zug161/Pompadour, Ricardo/Gus, and Fumai8/Baronesse) were genotyped using the DArT-seq™ platform (www.diversityarrays.com) as outlined by Kilian et al. (2012). DArT-seq identifies both co-dominant single-nucleotide polymorphisms (SNPs) and dominant Silico DArT markers characterised by “presence–absence” variants (PAVs), collectively referred to as DArT-seq markers (Raman et al., 2013).

### International panel marker filtering and population structure

The Barley GBS 1.0 platform DArT genotyping identified >13K polymorphic *in silico* DArT-seq markers for the international panel. The marker data were curated by removing markers that were heterozygous (≥10%), monomorphic, without mapped positions, and with minor allele frequencies (MAFs) <5%. Markers that failed to provide information (i.e., missing data ≥20%) were also removed. Finally, a total of 11,328 unique DArT-seq markers with map positions in the Barley Morex V1 genome assembly (Consortium IBGS, 2012) were selected for further analysis. Chromosome 2H had the highest marker saturation and chromosome 4H had the lowest, with 2,282 and 926 markers, respectively. The number of markers per chromosome is provided in **Supplementary File S3**, and genome coverage is visualised in **Supplementary File S4**. Genetic relationships among accessions were investigated using principal component analysis (PCA) performed in R (R Core Team, 2020). A genetic kinship matrix was calculated using the “synbreed” package from Wimmer et al. (2012), and the first three principal components were visualised as a biplot with individuals classified by continent of origin using “ggplot 2” from Wickham (2016).

### Genome-wide association mapping

Phenotypic data (BYR CI scores) from four environments were paired with genotypic data (11,328 DArT-seq markers) for GWAS of the international panel. GWAS was performed using a single-locus mixed linear model with the “rrBLUP” package (Endelman, 2011). Genetic control was investigated based on the quantile versus theoretic quantile (QQ) plots, and five principal components were included as fixed effects in the final model. Kinship relatedness (K) was accounted for in the GWAS linear mixed model through the covariance between lines as calculated with “synbreed”. No clustering by class was observed and was therefore not included as fixed in the model. The Manhattan plots derived from the GWAS showed that significant SNP markers had higher −log_10_(*p*) values than false discovery rate thresholds, suggesting strong marker-trait associations (**Supplementary File S4**). Significant marker-trait associations were determined using the threshold −log_10_(*p*) > 4 (significant at the 0.001% level). Marker-trait association was only considered a QTL if −log_10_(*p*) > 4 significance was detected in at least one environment and two or more markers associated with the trait. Markers positioned within 5 cM on the Barley Morex V1 genome assembly (Consortium IBGS, 2012) were considered part of the same QTL cluster and the most strongly associated marker presented as the QTL “peak”. QTLs detected in the international panel follow the naming convention *RpshQ.GWA.ChrH.X*, where *Chr* is chromosome, *H* stands for *Hordeum*, and *X* is the identifier. The allele for resistance (phase) was determined for each marker based on the mean effect on phenotype (**Supplementary File S5**). Linkage disequilibrium (LD) analysis of the DArT-seq markers linked with QTL was performed in R using package snpStats (Clayton, 2023) representing pairwise LD as R^2^ between pairs of markers (**Supplementary File S6**).

### Biparental population marker filtering and parental encoding

The Barley GBS 1.0 platform DArT genotyping for service returned between 6 and 27K markers depending on the population (Pompadour/Zhoungdamei, 14,661 markers; CI9214/Baudin, 18,628 markers; Zug161/Pompadour, 21,164 markers; Ricardo/Gus, 27,898 markers; and Fumai8/Baronesse; 6,511 markers). Markers were filtered to only those that carried a different marker phase in parental genotypes and progeny encoded to identify which parent contributed the region (raw binary genotype calls were converted to “A” and “B” genotype calls based on parental phasing) for final parental encoded sets (Pompadour/Zhoungdamei, 11,793; CI9214/Baudin, 9,456 markers; Zug161/Pompadour, 3,346 markers; Ricardo/Gus, 3,225 markers; and Fumai8/Baronesse, 6,299 markers).

### Marker frequency analysis of biparental populations

The frequency of the alleles carried by resistant progeny was compared with the frequency of the alleles carried by susceptible progeny in each of the parental encoded RIL sets. A discriminant value reflecting the level of allelic discrimination between the two classes was calculated for each marker (Wenzl et al., 2006, 2007). A simple chi-squared test was performed at each marker to detect significant discrimination between observed and expected allele frequencies. A differential threshold of >0.1 discriminant value was used to consider a marker significantly associated with a trait, which was calculated to have a <0.3% probability of associating an allele with resistance by chance. Greater than 1 significantly associated marker positioned within 5 cM on the Barley Morex V1 genome assembly (Consortium IBGS, 2012) was considered a QTL and the most strongly associated marker presented as the QTL “peak”. The parent contributing to the allele for resistance was determined for each marker (**Supplementary File S7**). Naming QTL detected in the biparental QTL mapping families follows the convention *RpshQ.Donor.ChrH*, where *Donor* is the parental allele genotype and *Chr* is the chromosome.

### Marker projection and visualisation

Sequences associated with significant markers were projected onto the Barley Morex V2 genome assembly (Monat et al., 2019) using GrainGenes (Yao et al., 2022) BLASTN 2.12.0+ server (Priyam et al., 2019) with an evalue of 1e−05. Regions of interest were visualised on the Barley Morex V2 genome assembly (Monat et al., 2019) using MapChart 2.2 (Wageningen UR; Voorrips, 2002).

### Development of markers and saturation of *RpshQ.Bau.1H* region

A 1.46-Mbp (8.14–9.60) genomic region identified on chromosome 1H through GWAS and biparental mapping of CI9214/Baudin RIL population based on the Morex reference v2.0 was enriched with both microsatellite and KASP markers. This genomic region was targeted because it was commonly detected in GWAS and three of the five biparental populations, and additionally, plant defence resistance genes were also identified in this region.

Closely linked DArT-seq markers for the chromosome 1H region harbouring *RpshQ.Bau* were subjected to BlastN search in the IPK barley blast server (https://galaxy-web.ipk-gatersleben.de/) against barley Morex reference genome v2.0 (2019). Discovered contigs were screened using the Simple Sequence Repeat Identification Tool (SSRIT) program (http://www.gramene.org/gramene/searches/ssrtool), and contigs that included short tandem repeats were used to design 17 simple sequence repeat (SSR) markers using the BatchPrimer3 (https://probes.pw.usda.gov/cgi-bin/batchprimer3/batchprimer3.cgi) program. The 17 SSR primers were tested on CI9214 and Baudin for parental polymorphism using the PCR assay described in Chhetri et al. (2016). The PCR products were separated and visualised on high-resolution capillary electrophoresis QIAxcel Advanced System, and gel data were analysed using QIAxcel Screen Gel software. The polymorphic markers were symbolised with the prefix (*sun* = Sydney University) followed by donor parent and chromosome number.

To develop KASP markers, associated SNPs identified in the target region of *RpshQ.Bau.1H* were used directly to generate two allele-specific forward primers and one common reverse primer, or vice versa using Batch Primer 3 (https://probes.pw.usda.gov/cgi-bin/batchprimer3/batchprimer3.cgi). Twenty KASP markers were developed and examined on parental DNA samples including three resistant and susceptible lines from the population using the Bio-Rad CFX96 Touch™ Real-Time PCR Detection System as described by Chhetri et al. (2016).

Chi-squared analysis was used to verify goodness-of-fit for observed segregation to expected marker genetic ratios. Markers that were polymorphic between resistant and susceptible bulks and parents were mapped in the CI9214/Baudin RIL population to saturate the chromosomal region encompassing *RpshQ.Bau.1H*. A genetic linkage map was created using QTXb20 software (Manly et al., 2001), and a recombinant fraction (RF) was converted to centimorgan (cM) using the Kosambi map function (Kosambi, 1943). The resulting map spanned 17.1 cM, corresponding to 1.49 Mb in the Barley Morex V2 genome. A logarithm of odds (LOD) score of ≥3 was applied to ascertain the significance of genetic linkages between molecular markers and the resistance locus. MapChart version 2.32 software (Voorrips, 2002) was used for generating the final map.

## Results

### Disease assessment of the international panel

**Figure 1:**
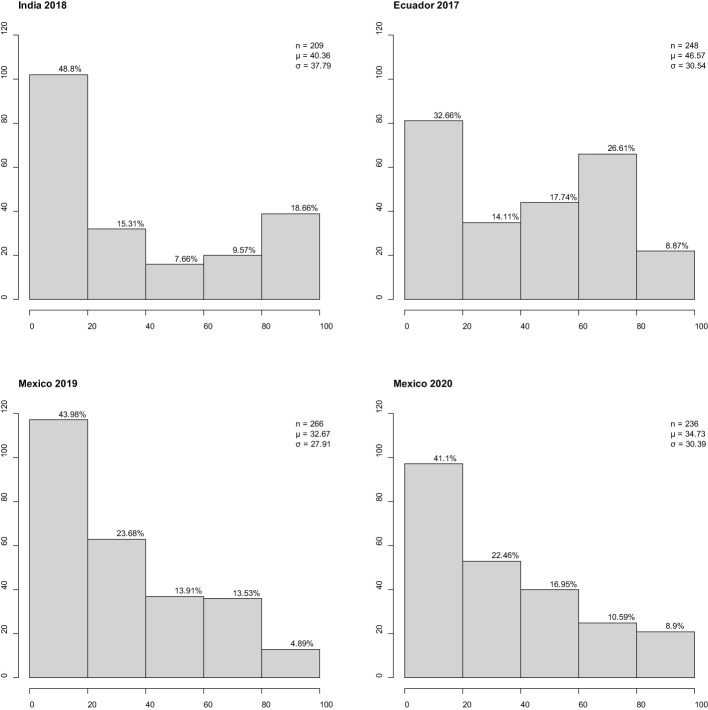
Frequency distributions of barley stripe rust coefficient of infection observed in the international panel at three international sites in four different years. x-Axis is disease response range, and y-axis is number of individuals. Classification of resistance spectrum: resistant (0–20), moderately resistant (21–40), moderately resistant to moderately susceptible (41–60), moderately susceptible (61–80), and susceptible (81–100). Correlation coefficient r^2^ (Ecuador 2017, India 2018) = 0.47***, r^2^ (Ecuador 2017, Mexico 2019) = 0.57***, r^2^ (Ecuador 2017, Mexico 2020) = 0.64***, r^2^ (India 2018, Mexico 2019) = 0.42***, r^2^ (India 2018, Mexico 2020) = 0.46***, and r^2^ (Mexico 2019, Mexico 2020) = 0.69***. Percentage of individuals in class provided above bar. Population size, mean, and standard deviation are provided for each site. *** Significant at 0.01% level.

Stripe rust established well in all four environments (Ecuador, Mexico x2, and India). The disease response (CI) for adult plants assessed across the environments ranged from 0 to 100 (**Figure 1**). All environments had low-to-moderate positive correlation coefficient for international panel CI: Mexican sites were moderately correlated with each other (r = 0.69***); the Ecuadorian site was moderately correlated with Mexican sites [2019 (r = 0.57***) and 2020 (r = 0.64***)]; the Indian sites had a low correlation with those in Mexico [2019 (r = 0.42***) and 2020 (r = 0.46***)] or Ecuador (r = 0.47***) sites. All correlation coefficients were statistically significant (p < 0.0001), indicating confidence in the correlation presented. At both Mexican sites, CI frequency distribution was skewed towards resistance; at the Ecuadorian site, CI frequency distribution was non-symmetric bimodal, skewed towards resistance, and had a secondary peak at moderate susceptibility; and at the Indian site, CI frequency distribution was “u”-shaped and skewed towards resistance.

### Population structure of international panel and linkage disequilibrium

**Figure 2:**
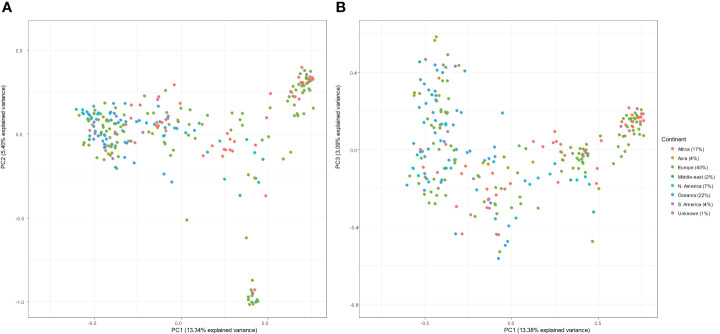
Principal component analysis of the kinship matrix visualising the genetic relationships between 266 lines. The figure on the left **(A)** represents the first principal component (PC1; x-axis) and the second principal component (PC2; y-axis), and the figure on the right **(B)** represents PC1 (x-axis) and the third principal component (PC3; y-axis). In both plots, genotypes are coloured according to continent.

Principal component analysis of genetic similarity was performed on the international panel filtered set of 11,328 DArT-seq markers. There was no structured clustering in terms of continents or country of origin observed across the genetic data of the genotypes. The PC1 and PC2 explained the accumulated genotypic variation of 13.34% and 5.40%, respectively (**Figure 2**).

The LD analysis of DArTs linked with QTLs detected via GWAS revealed varying degrees of pairwise LD, represented as R^2^ values between marker pairs (**Supplementary File S6**). A cluster of *RpshQ.GWA.chr1.1* markers, including 3985398, 3985766, 3985777, 3987315, 3913498, and 3270251, displayed high LD with each other (R^2^ = 0.75 to 1). Markers associated with *RpshQ.GWA.chr1.2*, such as 3267646, 3267658, and 3268171, showed strong linkage with each other (R^2^ = 0.78 to 0.9) but not with other markers, indicating an independent QTL. Markers linked to *RpshQ.GWA.chr7*, such as 3432111 and 3271387, showed moderate-to-strong LD with each other (R^2^ = 0.73), also indicating an independent QTL. Markers that are not part of the main *RpshQ.GWA.chr1.1* cluster, such as 3259989, 3255064, 3263243, and 3910455, as well as markers associated with *RpshQ.GWA.chr1.3*, appear to be moderately linked with each other.

### GWAS of international panel

Analysis of individual stripe rust response data detected a total of four significant QTLs at −log_10_(*p*) ≥ 4 in at least one environment (**Table 1**). Markers significantly associated with disease response were identified on chromosomes 1H (three QTLs) and 7H (one QTL). The QTL *RpshQ.GWA.1H.1* was detected in two environments (Ecuador and India) at log_10_(*p*) ≥ 4 and in one environment (Mexico 2020) at log_10_(*p*) ≥ 3. *RpshQ.GWA.1H.2* was environment or race specific to India 2018. *RpshQ.GWA.chr1H.3* and *RpshQ.GWA.chr7H* were detected only at the Mexico_2019 site. The number of markers contributing to the QTL and peak DArT clone ID are presented in **Table 1**, and −log_10_(*p*) and effects (ranging from −15.33 to 31.61) are presented in **Supplementary File S5**. *RpshQ.GWA.1H.1* was considered the key candidate, as this region contained 16 significantly associated markers within a 9.07-Mbp interval (1.33–10.40 on chromosome 1H) and was stable across all environments assessed. All other QTLs contained two to three markers. Three other loci were detected on chromosomes 2H, 3H, and 6H at log_10_(*p*) ≥ 4 but were associated with only a single marker.

**Table 1:** QTLs and markers associated with resistance to stripe rust detected under four environments in an association mapping panel (n = 266).

QTL name	Chr*	Position (Mbp)	Environments detected	No. of markers contributing	Peak marker/clone ID	Peak marker position (Mbp)
*RpshQ.GWA.chr1H.1*	1	1.33–10.40	Ecuador_2017, India_2018	16	3429708	8.15
*RpshQ.GWA.chr1H.2*	1	28.55–28.60	India_2018	3	3267646	28.55
*RpshQ.GWA.chr1H.3*	1	495.20–495.22	Mexico_2019	2	3263243	495.22
*RpshQ.GWA.chr7H*	7	508.05–517.73	Mexico_2019	2	3432111	508.05

Clone ID, sequence, LOG, and effect are provided in **Supplementary File S5**.

QTLs, quantitative trait loci.

* Chr morex_rev2_2019.

### Disease assessment of RILs

**Figure 3:**
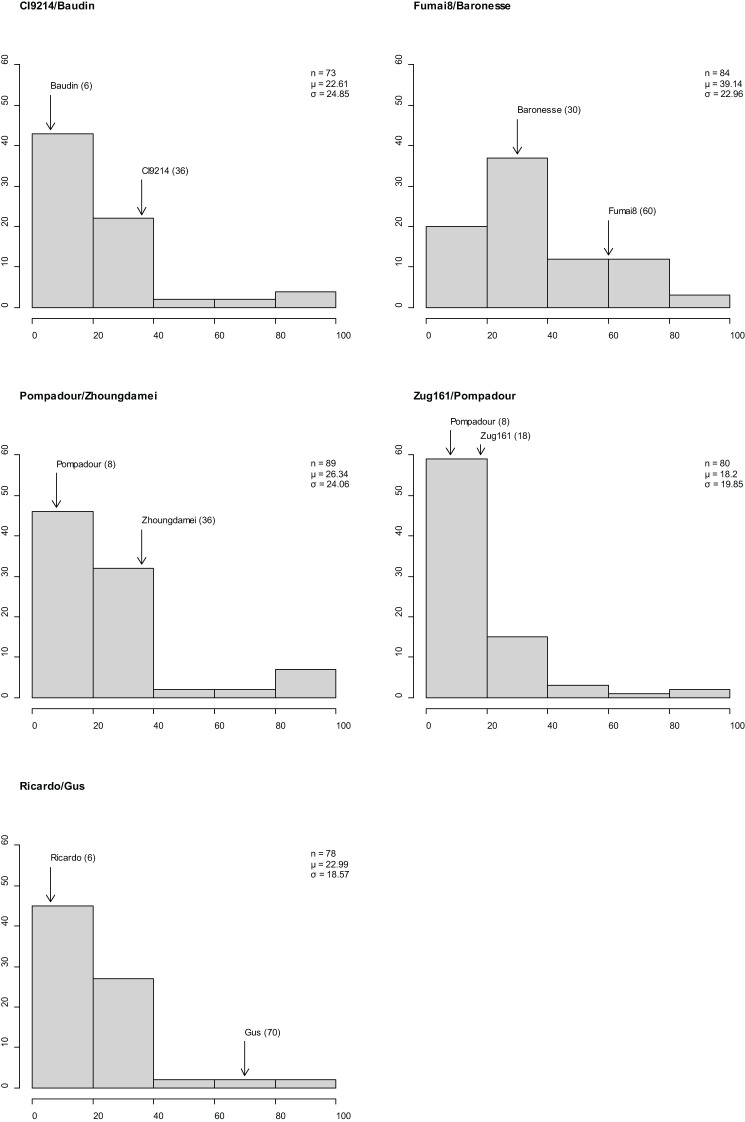
Frequency distributions of barley stripe rust coefficient of infection in five recombinant inbred line (RIL) population observations in Mexico. x-Axis is disease response range, and y-axis is number of individuals. Classification of resistance spectrum: R = resistant (0–20), MR = moderately resistant (21–40), MR-MS = moderately resistant to moderately susceptible (41–60), MS = moderately susceptible (61–80), and S = susceptible (81–100). Population size, mean, and standard deviation are provided for each population. Coefficient of infection (CI) of parental line is indicated above bar.

Stripe rust established well in Mexico 2020 (susceptible spreaders reached 90–100S evenly throughout the nursery), segregation for resistance and susceptibility was observed in the five RIL populations, and CI distributions varied across populations and parents (**Figure 3**). The disease response (CI) for adult plants ranged from 0 and 100 in populations CI9214/Baudin, Pompadour/Zhoungdamei, and Zug161/Pompadour, 12 to 100 in Fumai8/Baronesse and 0 to 90 in Ricardo/Gus, with all populations skewed towards resistance. Differences in parental scores were observed for all mapping populations: CI9214 (36)/Baudin (6), Pompadour (8)/Zhoungdamei (36), Zug161 (18)/Pompadour (8), Fumai8 (60)/Baronesse (30), and Ricardo (6)/Gus (70).

### Biparental marker coverage and mapping of RILs

Populations Pompadour/Zhoungdamei and CI9214/Baudin had the highest marker coverage on chromosome 2H and the lowest on 4H. Marker density in Zug161/Pompadour was the highest on 5H and the lowest on 1H. Marker coverage in Ricardo/Gus was the highest on 7H and the lowest on 4H. Marker diversity in Fumai8/Baronesse was the highest on 7H and the lowest on 1H. On average, coverage was the highest on 5H (1,286 markers), 2H (1,126 markers), and 7H (1,119 markers) and the lowest on 4H (656 markers) and 1H (700 markers). The number of markers per chromosome in each biparental population is provided in **Supplementary File S3**.

**Figure 4:**
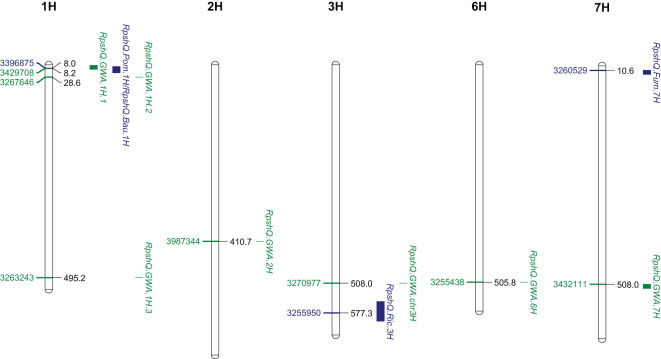
Genomic location of three significant quantitative trait loci (QTLs) from four families (*Rpsh_QRic*, *Rpsh_QPom*, *Rpsh_QFum*, and *Rpsh_QBau*) and genome-wide association study (GWAS) panel associated with resistance to barley yellow rust and position of peak markers linked to each of the QTL. Scale indicated Mbp on Barley Morex V2 genome assembly (Monat et al., 2019).

Mapping of the five RIL populations detected stripe rust resistance QTL on chromosomes 3H and 4H (Ricardo/Gus); 1H, 2H, 5H, and 7H (Pompadour/Zhoungdamei); 1H, 3H, 4H, and 7H (Fumai8/Baronesse); 1H, 4H, 5H, and 7H (CI9214/Baudin); and 1H and 5H (Zug161/Pompadour) (**Supplementary File S7**). Among these genomic regions, four resistance loci (*Rpsh_QRic.3H*, *Rpsh_QPom.1H*, *Rpsh_QFum.7H*, and *Rpsh_QBau.1H*) were the most significant in terms of maximum marker contribution (**Table 2**). *Rpsh_QPom.1H* was contributed by the resistant parent Pompadour in populations Pompadour/Zhoungdamei and Zug161/Pompadour with associations of 18 and 17 markers, respectively. *Rpsh_Qric.3H*, *Rpsh_QBau.1H*, and *Rpsh_QFum.7H* were contributed by parents Ricardo, Baudin, and Fumai8 with association of 32, 35, and 24 markers, respectively. The estimated phenotypic contribution (R^2^) of these four QTLs ranged from 5.2% to 35.0% depending upon the mapping population (**Table 2**). *Rpsh_QPom.1H* and *Rpsh_QBau.1H* were detected in the same genomic region in three independent populations, are likely the same, and are hence referred to as *RpshQ.Pom/Bau.1H*. Closely linked markers for each of these QTLs were identified; their positions and details are presented in **Table 2**; **Supplementary File S7**, and **Figure 4**.

**Table 2:** Position of the key genomic regions detected and peak markers closely linked to QTLs conferring resistance to barley yellow rust in five RIL populations (Ricardo/Gus, Pompadour/Zhoungdamei, Fumai8/Baronesse, CI9214/Baudin, and Zug161/Pompadour).

QTL name	Chr*	Position (Mbp)	No. of markers contributing	Peak marker/clone ID	Peak marker position (Mbp)	Phenotypic contribution R^2^ (%)
*RpshQ.Pom/Bau.1H*	1	3.91–18.59	70^A^	3396875	8.01	5.2^1^/35.0^2^/10.3^3^
*RpshQ.Ric.3H*	3	550.68–596.60	32^B^	3255950	577.32	9.6^4^
*RpshQ.Fum.7H*	7	9.93–19.93	24^C^	3260529	10.55	6.2^5^

Clone ID, sequence, and discriminant are provided in **Supplementary File S7**.

QTLs, quantitative trait loci; RIL, recombinant inbred line.

^A^ A total of 18 markers contributed by Pompadour (Pompadour/Zhoungdamei RIL population), 35 contributed by Baudin (CI9214/Baudin), and 17 contributed by Pompadour (Zug161/Pompadour).

^B^ Contributed by Ricardo (Ricardo/Gus).

^C^ Contributed by Fumai8 (Fumai8/Baronesse).

^1^ Pompadour/Zhoungdamei.

^2^ CI9214/Baudin.

^3^ Zug161/Pompadour.

^4^ Ricardo/Gus.

^5^ Fumai8/Baronesse.

* Chr morex_rev2_2019.

### QTL co-location in GWAS and RILs

The QTL *RpshQ.GWA.chr1H.1* detected in GWAS of the international panel and the QTL *RpshQ.Pom/Bau.1H* contributed by Pompadour and Baudin (detected in Pompadour/Zhoungdamei, CI9214/Baudin, and Zug161/Pompadour) were the only QTLs that co-located across mapping studies. *RpshQ.GWA.chr1H.1* spanned 1.33–10.40 Mbp and *RpshQ.Pom/Bau.1H* spanned 3.91–19.59 on chromosome 1H of the Barley Morex V2 genome assembly (**Figure 4**).

### Saturation of *RpshQ.Pom/Bau.1H* and marker validation

**Figure 5:**
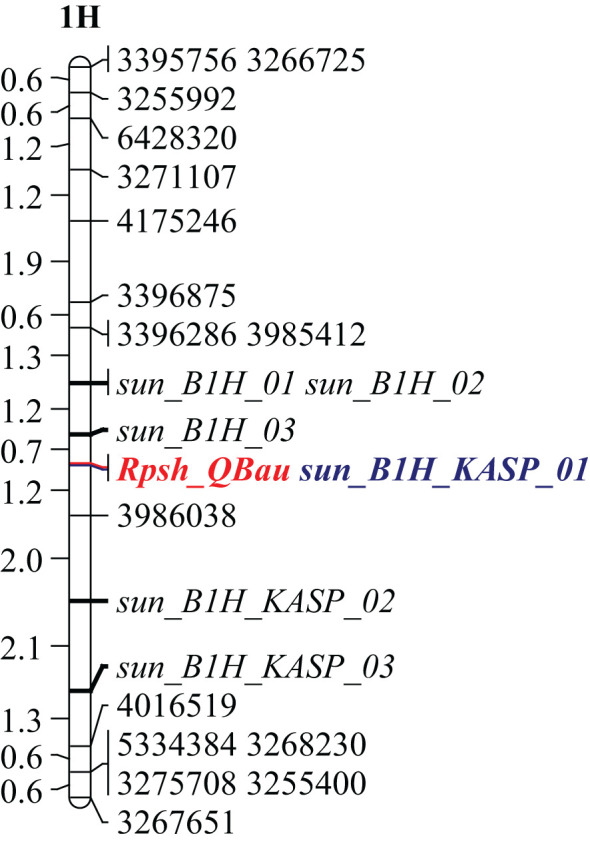
Genetic linkage map of chromosome 1H generated from recombinant inbred line (RIL) population CI9214/Baudin (linked marker co-segregating with *Rpsh_QBau* is highlighted in blue). Scale indicated in centi-Morgan on CI9214/Baudin genetic map.

Thirty-five markers in the vicinity of the *RpshQ.Pom/Bau.1H* resistance locus, spanning 8.14 to 9.60 Mbp on chromosome 1H of the Barley Morex V2 genome assembly, were targeted for saturating the genetic map of the CI9214/Baudin population. This region contributed significantly and stably (~35%) to phenotypic variation. We designed 17 SSR markers targeting this interval, with six showing polymorphism. Additionally, 20 KASP markers were designed, with eight being polymorphic. The linkage map, spanning a genetic distance of 17.1 cM and covering 1.49 Mb in the Barley Morex V2 genome, integrated four SSR markers and three KASP markers into the CI9214/Baudin genetic map (**Figure 5**). KASP marker *sun_B1H_KASP_01* co-segregated with *RpshQ.Pom/Bau.1H*, and SSR markers *sun_B1H_03* mapped 0.7 cM distal to *RpshQ.Pom/Bau.1H*. Marker *sun_B1H_KASP_01* was the most robust with clear allelic discrimination. The sequences of these markers are provided in **Table 3**. Marker *sun_B1H_KASP_01* was applied on 50 Australian barley genotypes/cultivars listed in **Supplementary File S2**. Marker genotyping showed well-defined allelic discrimination for the absence/presence of marker *sun_B1H_KASP_01* (**Supplementary File S8**) and six Australian genotypes (Baudin, Fathom, Flagship, Grout, Sakurastar, and Shepherd) were predicted to carry *RpshQ.Pom/Bau.1H*.

**Table 3:** Sequences of SNP and SSR markers mapped and developed for *Rpsh_1H*.

SNP markers	Allele 1 (A1) sequence (5′→3′)	Allele 2 (A2) sequence (5′→3′)	Common	SNPs
*Sun_B1H_KASP_01*	acaaccaaccaaccaagaaacG	acaaccaaccaaccaagaaacA	tccggttcagtggttgcatt	[G/A]
*Sun_B1H_KASP_02*	ctcgcatctaaggaaaatgcatG	ctcgcatctaaggaaaatgcatA	acccatagagctcggaacca	[G/A]
*Sun_B1H_KASP_03*	gctcatcaatgtaatcagagtgcC	gctcatcaatgtaatcagagtgcT	cggcaagaacaacgaaacct	[C/T]
**SSR markers**	**Forward sequence**	**Reverse sequence**		
*Sun_B1H_01*	aataccttgcaacaggtcgg	Cgtttcaggtgggtctgttt		
*Sun_B1H_02*	ctacgaccaccgtccagaat	Cgtttcaggtgggtctgttt		
*Sun_B1H_03*	acttacccggggtgaaacat	agatcgacgacggagatgag		

SNPs, single-nucleotide polymorphism; SSR, simple sequence repeat.

## Discussion

A systematic and efficient breeding approach to developing rust-resistant barley cultivars involves the discovery, characterisation, and mapping of new sources of resistance to diversifying the genetic base of resistance and the subsequent reliance on perfectly linked molecular markers for reliable and rapid selection. The studies reported here were conducted to understand the genetic architecture underlying resistance to *Psh* in a geographically diverse international barley panel, which had been previously mapped for response to leaf rust (Singh et al., 2018). Following the characterisation and mapping of resistance to *Psh* using extensive phenotyping at three international disease hotspots and GWAS of an international panel, we saturated and validated the underlying major QTL of interest using five biparental mapping populations segregating for stripe rust resistance and developed closely linked PCR-based markers for one of the most consistent and stable loci located on chromosome 1HS.

Our association studies on the international panel detected four QTLs (three on chromosome 1H and one on 7H) associated with resistance to stripe rust across or specific to four environments. Three additional loci were also detected on chromosomes 2H, 3H, and 6H but were associated with only a single marker. Several barley stripe rust GWASs have been conducted over the last 15 years, and over 50 QTLs have been reported across all seven chromosomes. Visioni et al. (2018) detected 15 adult growth-stage QTLs, and only QTL *APS_Dg_14_2* on chromosome 2H corresponded with a single marker 2H QTL detected in our study. This region also aligned with QTL *QPs.2H-1* detected by Vatter et al. (2018) in a HEB-25 population developed by Maurer et al. (2015). All QTLs detected in our study were distinct from the 25 loci identified in two other studies (Klos et al., 2016; Vatter et al., 2018) likely due to the use of divergent material.

It is not uncommon for GWAS to detect spurious marker-trait associations (false positives) and hence incorrect calling of a QTL (Prins et al., 2016; Kertho et al., 2015). To validate the GWAS results, we performed mapping on five biparental populations and in so doing detected co-location for the resistance loci *RpshQ.GWA.1H.1* and *RpshQ.Pom/Bau.1H*. QTL mapping on biparental mapping populations detected key genomic regions on chromosomes 1H (Pompadour/Zhoungdamei and Pompadour/Zug161, CI9214/Baudin), 3H (Ricardo/Gus), and 7H (Fumai8/Baronesse), which were designated *RpshQ.Pom/Bau.1H*, *Rpsh_QRic.3H*, and *Rpsh_QFum.7H*, respectively. *RpshQ.Bau.1H* was in the close vicinity of GWAS detected QTL *RpshQ.GWA.1H.1* (located 1.33–10.40 Mbp). *Rpsh_QFum.7H* on 7H was distinct and was not detected by GWAS. The findings reinforce that although GWAS provides superior resolution in detecting genomic locations, its statistical power nevertheless can be diluted by the inability to detect rare allele associations and genetic structure leading to false positives, as emphasised previously by Kertho et al. (2015). The failure to detect a genomic region corresponding to *Rpsh_QFum.7* in our GWAS may be attributed to a low frequency of this allele in the AM panel that we used or rare functional alleles that were removed during data curation.

The QTL *RpshQ.GWA.1H.1* detected by GWAS and the QTL *RpshQ.Pom/Bau.1H* detected using biparental mapping populations co-located on 1H were the most consistent and stable regions detected and are likely the same gene. Dracatos et al. (2019) also likely identified the same 1H QTL in a RIL population, Pompadour/Biosaline-19, based on screening at the same site in Mexico in 2015, with resistance also contributed by Pompadour. In addition, these authors also identified a corresponding 1H QTL in greenhouse seedling experiments using a Dutch *Psh* isolate. More recently, Bettgenhaeuser et al. (2021) also identified a 1H locus conferring resistance to the wheat-specialised formae speciales of stripe rust *tritici* (*Pst*) in a comparable region of 1H (8.92 cM) and designated it as *Rps7*. The genetic relationship between *Rps7* (effective to *Pst*) and the 1H QTL (effective to *Psh*) in our study cannot be established unless a joint segregation analysis and mapping is conducted with both *Pst* and *Psh* or with linked markers. Verhoeven et al. (2011) developed a series of stripe rust resistance near isogenic stocks (BIOSAN lines) and used line BCD_12 supposedly carrying a QTL for resistance to stripe rust originally identified in a Shyri/Galena population investigated by Toojinda et al. (2000). Their SNP consensus linkage map for the 1HS aligned with the Shyri/Galena linkage map, and SNP haplotypes of BIOSAN and BCD12 line established a major QTL peaking at a corresponding position of *RpshQ.GWA.1H.1* and *RpshQ.Bau.1H* detected in our study. The QTL *RpshQ.GWA.chr7* detected in this study collocated with *Rph_G_Q12*, a QTL associated with leaf rust resistance and reported previously in the same GWAS panel (Singh et al., 2018). Breeders prefer resistance genes that are linked to other disease resistance genes or are pleiotropic. Further studies will be useful to investigate and understand the genetic relationship between *RpshQ.GWA.chr7* and *Rph_G_Q12*.

The stable detection of *RpshQ.GWA.1H.1/RpshQ.Pom/Bau.1H* in this study and various previous studies suggest that this allele is quite common in international germplasm and that it is a locus of importance in conferring resistance to *Psh* on a global scale. We further saturated *RpshQ.Pom/Bau.1H*, placing it between 8.14 and 9.60 Mbp on 1HS in the Morex reference genome v.2 (Monat et al., 2019). The locus was flanked by SSR markers *sun_B1H_03* (0.7 cM proximal to *Rpsh_1H*) and *sun_B1H_KASP_02* (3.2 cM distal) and a developed KASP marker *sun_B1H_KASP_01* that co-segregated with *RpshQ.Pom/Bau.1H*. Within the targeted region of 8.14–9.60 Mbp on chromosome 1HS, 12 high-confidence genes or encode proteins were identified, including Leucine-tRNA ligase, disease resistance proteins, chymotrypsin inhibitors, NAD(P)-binding Rossmann-fold protein, and RING/U-box superfamily protein. These genes are functionally associated with disease resistance mechanisms and may play a role in the stripe rust resistance imparted by *RpshQ.Bau*.

The closely linked marker *sun_B1H_KASP_01* identified in this study is highly robust and can be used reliably for marker-assisted selection of *RpshQ.Pom/Bau.1H*. This marker, when tested on a set of 50 Australian barley cultivars, showed well-defined allelic discrimination. Six of the test cultivars (Baudin, Fathom, Flagship, Grout, Sakurastar, and Shepherd) were identified to carry *RpshQ.Pom/Bau.1H* based on the marker and recommended to breeders for further utilisation in breeding. The identified SNP marker and germplasm are of high importance, especially for countries like Australia where *Psh* is an exotic threat, and breeders can use the developed marker and source of resistance for anticipatory breeding and pyramiding of resistance genes for achieving durable stripe rust resistance in future cultivars.

## Data availability statement

The original contributions presented in the study are included in the article/**Supplementary Material**. Further inquiries can be directed to the corresponding author.

## Author contributions

DS: Conceptualization, Methodology, Writing – original draft, Writing – review & editing. LZ: Formal analysis, Writing – review & editing, Methodology. MC: Methodology, Writing – review & editing. PD: Writing – review & editing, Methodology. KF: Methodology, Writing – review & editing. SrB: Writing – review & editing, Methodology. RS: Writing – review & editing, Methodology. CB: Writing – review & editing, Methodology. PN: Writing – review & editing, Methodology. OG: Writing – review & editing, Methodology. SK: Writing – review & editing, Methodology. SuB: Writing – review & editing, Methodology. RP: Writing – review & editing, Funding acquisition.
