# Supplementary material for: Genetic mapping of stripe rust resistance in a geographically diverse barley collection and selected biparental populations
Source: Front Plant Sci. 2024 Jul 19;15:1352402. doi: 10.3389/fpls.2024.1352402 (PMC11299494; doi:10.3389/fpls.2024.1352402)
Supplement: Supplementary file 4 [file Table_4.docx]

**Supplementary file S4**: Manhattan plots representing markers associated with BYR resistance in international panel in four field environments: (A) Ecuador 2017 (B) India 2018 (C) Mexico 2019 and (D) Mexico 2020. The grey horizontal line represents a genome-wide significance threshold of log10(p) of 2 (>1%), the solid red horizontal line represents a genome-wide significance threshold of log10(p) of 3(>0.1%) and solid blue line represents a genome-wide significance threshold of log10(p) of 4(>0.01%). Density bands colour-coded to show genotypic distribution of 11,328 SNPs on the Barley Morex V1 genome assembly (Consortium IBGS 2012).


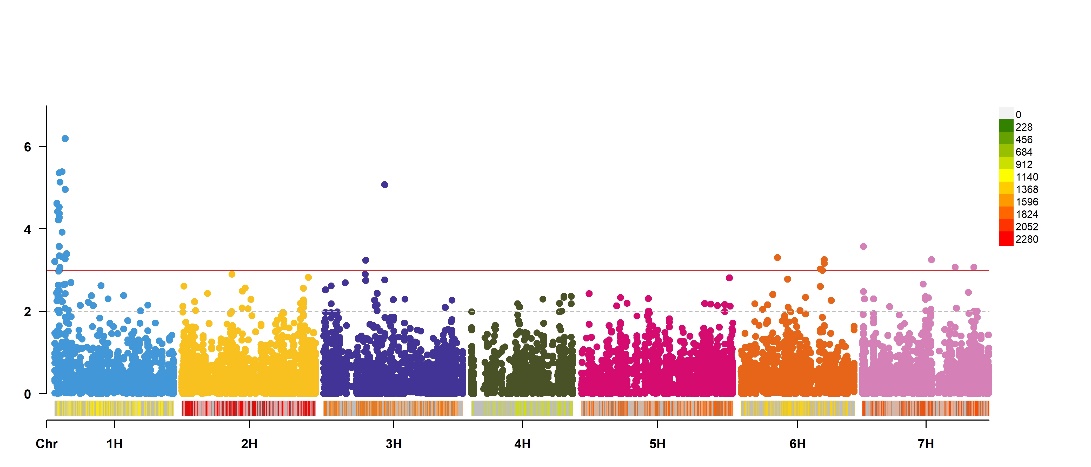


A


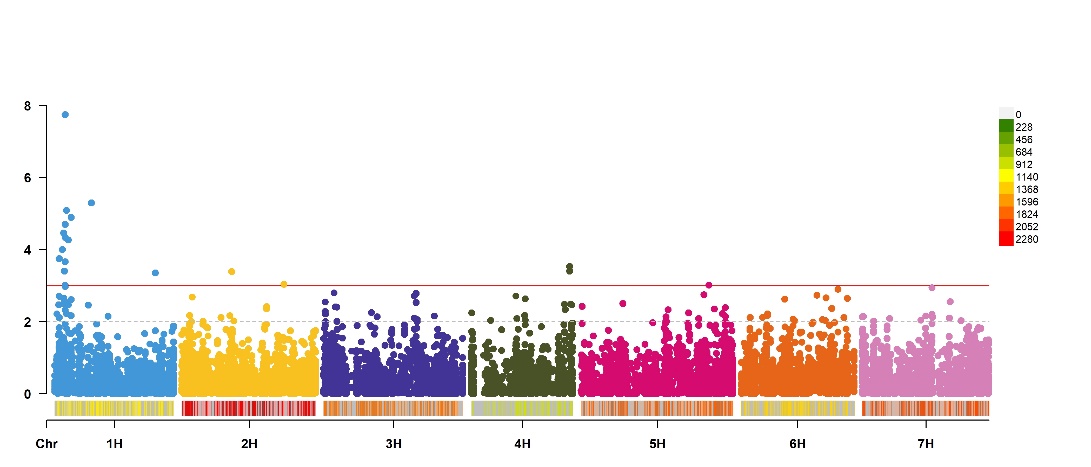


B


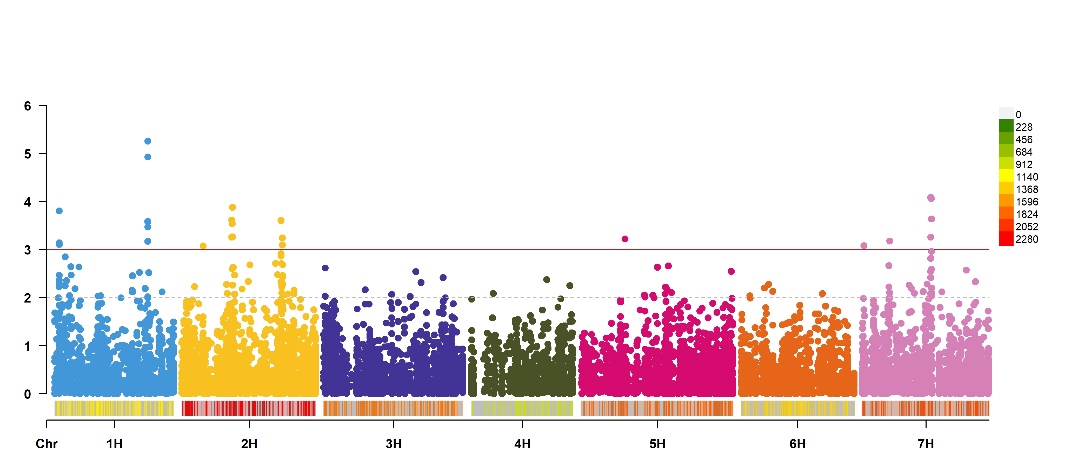


C


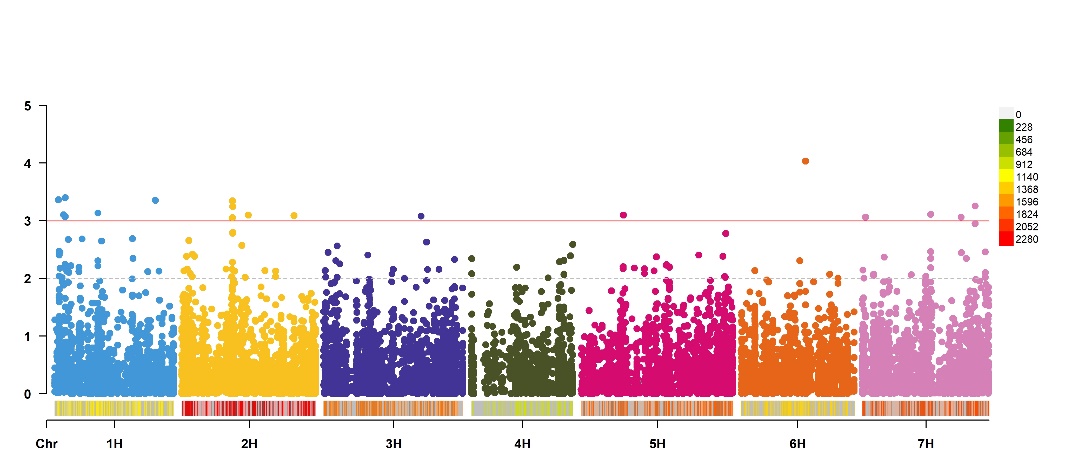


D
