# Supplementary material for: Genetic mapping of stripe rust resistance in a geographically diverse barley collection and selected biparental populations
Source: Front Plant Sci. 2024 Jul 19;15:1352402. doi: 10.3389/fpls.2024.1352402 (PMC11299494; doi:10.3389/fpls.2024.1352402)
Supplement: Supplementary file 6 [file Table_6.docx]

**Supplementary file S6.** Linkage disequilibrium (LD) analysis of the DArTs linked with QTL detected via GWAS performed on international panel. Heat map represents pairwise LD as R^2^ between pairs of markers.

| Clone ID |  | 3431212 | 3266635 | 3985963 | 3267658 | 3267646 | 3268171 | 3985777 | 3262034 | 3270251 | 3985766 | 3913498 | 3263243 | 3910455 | 3985398 | 3987315 | 3256503 | 3259989 | 3255064 | 3432135 | 3271033 | 3429708 | 3987344 | 3270977 | 3255438 | 3432111 |
| --- | --- | --- | --- | --- | --- | --- | --- | --- | --- | --- | --- | --- | --- | --- | --- | --- | --- | --- | --- | --- | --- | --- | --- | --- | --- | --- |
| 3266635 | RpshQ.GWA.chr1.1 | 0.06 |  |  |  |  |  |  |  |  |  |  |  |  |  |  |  |  |  |  |  |  |  |  |  |  |
| 3985963 | RpshQ.GWA.chr1.1 | 0 | 0.02 |  |  |  |  |  |  |  |  |  |  |  |  |  |  |  |  |  |  |  |  |  |  |  |
| 3267658 | RpshQ.GWA.chr1.2 | 0.01 | 0.04 | 0.01 |  |  |  |  |  |  |  |  |  |  |  |  |  |  |  |  |  |  |  |  |  |  |
| 3267646 | RpshQ.GWA.chr1.2 | 0.01 | 0.04 | 0.01 | 0.82 |  |  |  |  |  |  |  |  |  |  |  |  |  |  |  |  |  |  |  |  |  |
| 3268171 | RpshQ.GWA.chr1.2 | 0.01 | 0.05 | 0.01 | 0.9 | 0.78 |  |  |  |  |  |  |  |  |  |  |  |  |  |  |  |  |  |  |  |  |
| 3985777 | RpshQ.GWA.chr1.1 | 0 | 0.02 | 0.81 | 0.01 | 0.01 | 0.01 |  |  |  |  |  |  |  |  |  |  |  |  |  |  |  |  |  |  |  |
| 3262034 | RpshQ.GWA.chr1.1 | 0.05 | 0.23 | 0.04 | 0.19 | 0.21 | 0.2 | 0.05 |  |  |  |  |  |  |  |  |  |  |  |  |  |  |  |  |  |  |
| 3270251 | RpshQ.GWA.chr1.1 | 0 | 0.02 | 0.63 | 0.01 | 0.01 | 0.02 | 0.83 | 0.05 |  |  |  |  |  |  |  |  |  |  |  |  |  |  |  |  |  |
| 3985766 | RpshQ.GWA.chr1.1 | 0 | 0.02 | 0.77 | 0.01 | 0.01 | 0.02 | 0.89 | 0.05 | 0.73 |  |  |  |  |  |  |  |  |  |  |  |  |  |  |  |  |
| 3913498 | RpshQ.GWA.chr1.1 | 0 | 0.02 | 0.74 | 0.01 | 0.01 | 0.02 | 0.88 | 0.05 | 0.75 | 1 |  |  |  |  |  |  |  |  |  |  |  |  |  |  |  |
| 3263243 | RpshQ.GWA.chr1.3 | 0.01 | 0.09 | 0 | 0.03 | 0.03 | 0.06 | 0.01 | 0.22 | 0.01 | 0 | 0.01 |  |  |  |  |  |  |  |  |  |  |  |  |  |  |
| 3910455 | RpshQ.GWA.chr1.3 | 0.08 | 0.03 | 0.01 | 0.03 | 0.01 | 0.03 | 0.01 | 0.17 | 0.01 | 0 | 0 | 0.11 |  |  |  |  |  |  |  |  |  |  |  |  |  |
| 3985398 | RpshQ.GWA.chr1.1 | 0 | 0.02 | 0.71 | 0.01 | 0.01 | 0.02 | 0.91 | 0.05 | 0.9 | 0.81 | 0.82 | 0.02 | 0.01 |  |  |  |  |  |  |  |  |  |  |  |  |
| 3987315 | RpshQ.GWA.chr1.1 | 0 | 0.02 | 0.68 | 0.02 | 0.02 | 0.02 | 0.85 | 0.06 | 1 | 0.75 | 0.75 | 0.01 | 0.01 | 0.87 |  |  |  |  |  |  |  |  |  |  |  |
| 3256503 | RpshQ.GWA.chr1.1 | 0.07 | 0.06 | 0.02 | 0 | 0.03 | 0 | 0.02 | 0.14 | 0.02 | 0.02 | 0.02 | 0.12 | 0 | 0.02 | 0.02 |  |  |  |  |  |  |  |  |  |  |
| 3259989 | RpshQ.GWA.chr1.1 | 0.06 | 0.32 | 0.04 | 0.15 | 0.14 | 0.2 | 0.05 | 0.67 | 0.05 | 0.05 | 0.05 | 0.24 | 0.19 | 0.05 | 0.06 | 0.29 |  |  |  |  |  |  |  |  |  |
| 3255064 | RpshQ.GWA.chr1.1 | 0.06 | 0.3 | 0.05 | 0.15 | 0.16 | 0.19 | 0.05 | 0.71 | 0.06 | 0.06 | 0.06 | 0.26 | 0.21 | 0.06 | 0.06 | 0.3 | 0.94 |  |  |  |  |  |  |  |  |
| 3432135 | RpshQ.GWA.chr1.1 | 0.47 | 0.11 | 0 | 0.01 | 0 | 0.01 | 0 | 0.02 | 0 | 0 | 0 | 0.02 | 0.02 | 0 | 0 | 0.06 | 0.05 | 0.04 |  |  |  |  |  |  |  |
| 3271033 | RpshQ.GWA.chr1.1 | 0.11 | 0.03 | 0.02 | 0.04 | 0.04 | 0.04 | 0.02 | 0.2 | 0.02 | 0.02 | 0.02 | 0.09 | 0.04 | 0.02 | 0.02 | 0.1 | 0.25 | 0.32 | 0.14 |  |  |  |  |  |  |
| 3429708 | RpshQ.GWA.chr1.1 | 0.12 | 0.02 | 0.02 | 0.03 | 0.04 | 0.04 | 0.02 | 0.22 | 0.02 | 0.02 | 0.02 | 0.08 | 0.09 | 0.02 | 0.02 | 0.09 | 0.26 | 0.34 | 0.13 | 0.9 |  |  |  |  |  |
| 3987344 | RpshQ.GWA.chr2 | 0 | 0.02 | 0.69 | 0.01 | 0.01 | 0.01 | 0.9 | 0.05 | 0.83 | 0.8 | 0.8 | 0.02 | 0.01 | 0.91 | 0.78 | 0.02 | 0.05 | 0.05 | 0 | 0.02 | 0.02 |  |  |  |  |
| 3270977 | RpshQ.GWA.chr3 | 0.02 | 0.13 | 0.1 | 0 | 0.03 | 0.01 | 0.13 | 0.31 | 0.13 | 0.1 | 0.09 | 0.19 | 0.15 | 0.14 | 0.15 | 0.15 | 0.33 | 0.36 | 0.03 | 0.12 | 0.13 | 0.13 |  |  |  |
| 3255438 | RpshQ.GWA.chr6 | 0.03 | 0.1 | 0.04 | 0.06 | 0.06 | 0.12 | 0.07 | 0.4 | 0.07 | 0.06 | 0.06 | 0.27 | 0.14 | 0.07 | 0.1 | 0.21 | 0.44 | 0.47 | 0.03 | 0.16 | 0.16 | 0.06 | 0.47 |  |  |
| 3432111 | RpshQ.GWA.chr7 | 0.01 | 0.06 | 0.03 | 0 | #### | 0.01 | 0.06 | 0.15 | 0.04 | 0.03 | 0.03 | 0.02 | 0.02 | 0.04 | 0.04 | 0.07 | 0.15 | 0.17 | 0.01 | 0.01 | 0.01 | 0.04 | 0.07 | 0.22 |  |
| 3271387 | RpshQ.GWA.chr7 | 0.01 | 0.03 | 0.04 | 0.01 | 0 | 0 | 0.06 | 0.11 | 0.05 | 0.04 | 0.03 | 0.01 | 0.01 | 0.05 | 0.04 | 0.05 | 0.11 | 0.12 | 0.01 | 0 | 0 | 0.04 | 0.12 | 0.2 | 0.73 |
