# Supplementary material for: Genetic mapping of stripe rust resistance in a geographically diverse barley collection and selected biparental populations
Source: Front Plant Sci. 2024 Jul 19;15:1352402. doi: 10.3389/fpls.2024.1352402 (PMC11299494; doi:10.3389/fpls.2024.1352402)

**Supplementary file S8.** Allelic discrimination of KASP markers *sun_B1H_KASP_01* displaying *RpshQ.Bau* linked 'G' allele (positive, orange) and 'A' allele (negative, blue).


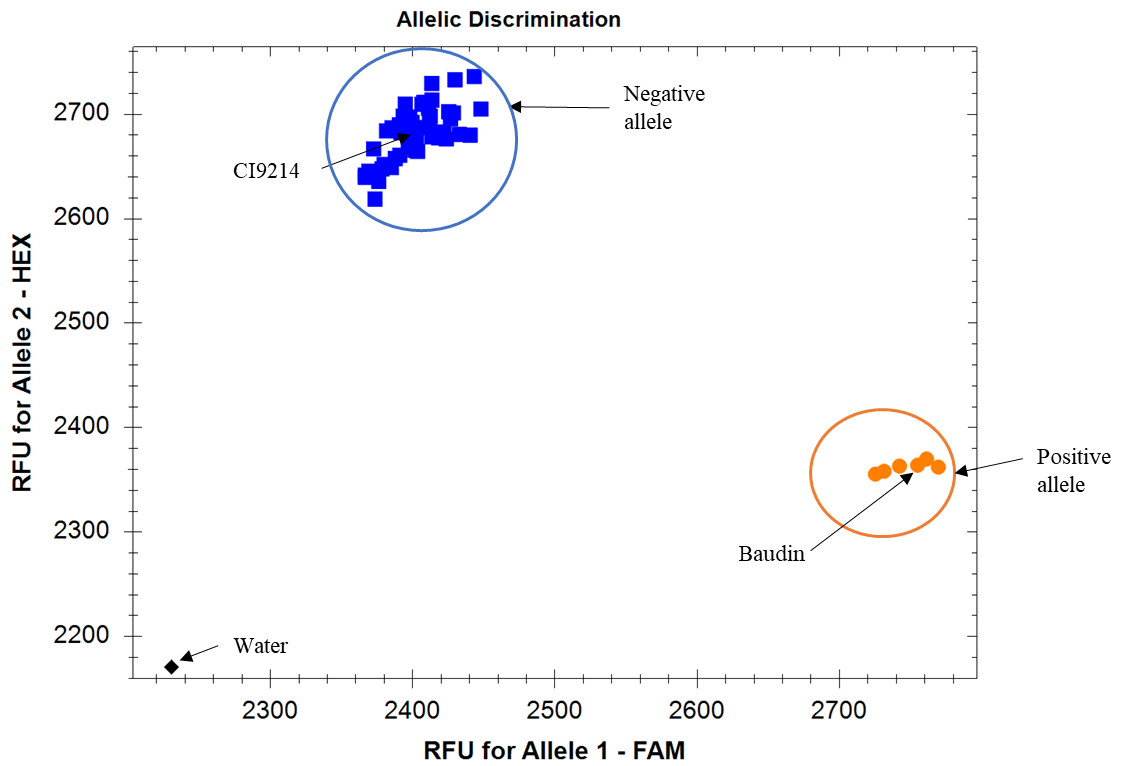

Supplement: Supplementary file 8 [file Table_8.docx]
